# A qualitative research study of primary care physicians’ views of telehealth in delivering postnatal care to women

**DOI:** 10.1186/s12875-022-01813-9

**Published:** 2022-08-13

**Authors:** Zhimin Poon, Ngiap Chuan Tan

**Affiliations:** 1 SingHealth Polyclinics, 167, Jalan Bukit Merah, Connection One, Tower 5, #15-10, Singapore, 150167 Singapore; 2 SingHealth-Duke NUS Family Medicine Academic Clinical Program, Singapore, Singapore

**Keywords:** Primary care physician, Postpartum care, Mother, Healthcare system, Telehealth, Telemedicine

## Abstract

**Background:**

The postpartum period is a critical time for women to optimise their physical and mental health. Primary care physicians (PCP) often manage postpartum women in the community setting after uneventful births. However, women encounter difficulties accessing care before and after their conventional 6-week physical review. Telehealth-based interventional studies have demonstrated their successful applications in several areas of postpartum care but is not widely adopted. The study aimed to explore the PCPs’ views on their acceptability and perceived barriers of telehealth in delivering postpartum care to women in primary care.

**Methods:**

Twenty-nine PCPs participated in eleven in-depth interviews and four focus group discussions for this qualitative study conducted in Singapore. The purposively sampled PCPs had varied demographic background and medical training. Two investigators independently coded the audited transcripts. Thematic content analysis was performed using the codes to identify issues in the pertaining to the perceived usefulness, ease of use and attitudes towards telehealth in postpartum care as described in the “Telehealth Acceptance Model” framework.

**Results:**

Most PCPs perceived usefulness and ease of use of video consultation in delivering postpartum care. They recognised telehealth service to complement and support the current face-to-face postpartum care amidst the pandemic. However, training, leadership support, organizational infrastructure, healthcare financial policy and personal demographic profile influence their acceptance of a new care model for postnatal mothers.

**Conclusion:**

Addressing the barriers and strengthening the facilitators will enhance PCPs’ acceptance and utilisation of the proposed hybrid (telehealth and in-person) postnatal care model for mothers.

**Supplementary Information:**

The online version contains supplementary material available at 10.1186/s12875-022-01813-9.

## Background

The postpartum period, defined as the 12 weeks following childbirth is a critical period for women. In this 4^th^ trimester, both mother and child have multiple medical and social needs [1]. The World Health Organization and multiple national clinical guidelines recommend comprehensive assessment of both women and child, which includes the physical, psychological, and social health of the postpartum women, their breastfeeding practices, nutrition advice, family planning, and child growth in the postpartum visit [2, 3]. Traditionally, the postpartum review scheduled at the 6-week mark often leaves mothers without an appointed medical provider to consult when they meet with difficulties in the early postpartum period [4]. During this time, mothers face several obstacles such as pain, postpartum bleeding, breastfeeding and care for their neonates. Adjusting to these new changes can often affect their emotional wellbeing [5].

There are approximately 39,000 multi-ethnic Asian women requiring postpartum care annually in Singapore [6]. Majority receive antenatal care from obstetricians in the tertiary setting. Midwifery support is limited in primary care practices as childbirth is medicalised and shifted to obstetrician-led hospital care in highly urbanised Singapore [7, 8]. Midwives mainly assist in delivery in tertiary setting and guide mothers in antenatal, intrapartum and postnatal care. Following childbirth, women with no intrapartum complications are discharged from hospitals within 2 to 3 days [9]. They then undergo a routine six-week physical postpartum review with their primary care physician (PCP) or obstetrician with no formal appointed care provider in between. Ong SF et al. highlighted the lack of clinician support and advice to primiparous mothers who experienced difficulty in achieving exclusive breastfeeding. Some of these mothers also had difficulty accessing a healthcare provider after discharge and felt apprehensive in contacting a health care provider for issues deemed “non-medical” such as infant care skills [10]. In another study, local PCPs reported fragmented postpartum support services and inadequate consultation time as barriers in providing optimal postpartum care to mothers [11].

The current COVID-19 pandemic further hinders access to postpartum care [12]. Mothers may defer their in-person postnatal review due to perceived risk of infection in community healthcare facilities. One solution to meet their postnatal care needs is to deliver it over a remote but secured platform. Telehealth refers to the systematic provision of healthcare services over physically separate environments via Information and Communications Technology (ICT) [13]. Telehealth has been increasingly deployed to provide clinical care during the pandemic [14]. Powell RE et al. showed patients’ perceived convenience and efficiency of telehealth-based services even before the onset of the pandemic [15]. Telehealth-based interventional studies have also demonstrated their successful applications in promoting early postpartum weight loss, breastfeeding exclusivity, the screening and follow up for postpartum depression [16–18].

Nonetheless, Powell RE et al. also highlighted patients’ concerns about the privacy and limitations of telehealth such as the inability of physicians to conduct physical examinations [15]. In the local community, telehealth is largely used by PCPs to serve patients with stable long-term diseases such as dyslipidaemia and hypertension, in which the need for physical examination is minimal [19]. However, its extension for PCPs to support postpartum care remains unclear. Poon Z et al. have already highlighted pertinent issues faced by PCPs in delivering in-person postnatal care [11]. Additional hurdles can be expected in transforming the postnatal care to a virtual care model. It is crucial to gather PCPs’ perspectives in order to design a dedicated telehealth service that focuses on the multiple needs of the postpartum mothers. The acceptability and confidence of PCPs in managing postnatal mothers over telehealth and their perceived barriers in the existing healthcare system and infrastructure have yet to be examined in planning the development of such services.

Hence, this study aims to explore the views and concerns among PCPs on their acceptability and perceived barriers of telehealth in delivering postpartum care to women in primary care.

## Methods

### Study design

A qualitative research method was employed [20] in order to deep dive into the interplay of factors influencing the perspectives of PCPs to deploy telehealth to support postpartum care delivery to women. Both focused group discussions (FGD) and in-depth interviews (IDI) were conducted to gather such qualitative data. FGDs were carried out first to gather the range in opinions and observe the exchange of viewpoints among the PCPs during their group discussions. PCPs of similar level of postgraduate training were grouped together for the FGDs to encourage them to discuss about the topic without reservations. A semi-structured interview guide was developed to facilitate the interviews. IDIs were then carried out to clarify and affirm the themes demonstrated in the FGDs.

### Theoretical framework

Based on a recent review on physicians’ adoption of mobile health tools, the Technology Acceptance Model (TAM) is commonly used to frame and describe their utility behaviour [21]. This comprehensive model [22] covers social and personal influences, data related concerns, workflow related issues and policy and organisational factors. The TAM was therefore selected as the theoretical framework to guide the conduct and analysis of the study. Video consultation over a secured remote platform is the focus of the discussion among the PCPs over their perceived usefulness, concern and ease of use of this technological application to deliver postpartum care.

### Site

The interviews were conducted at a public primary care clinic located in northeast Singapore. It mainly serves an estate populated with young families [23]. Face-to-face interviews were converted to digital interviews via the online teleconferencing platform, Zoom® due to restrictions during the COVID-19 pandemic in Singapore.

### Period of study

The study was conducted between February to October 2020 amidst the COVID-19 pandemic.

### Researcher characteristics

The study team comprised of one male and two female PCPs and a female nurse manager practising in SingHealth Polyclinics, a public primary care institution in Singapore. The female study team members had experienced childbirth and received postpartum care in Singapore.

### Study population

The study team interviewed multi-ethnic Asian PCPs from both the public and private general practices in the local dual primary healthcare system. They self-reported to provide care to postpartum women in their practices.

### Recruitment

Invitations were extended to the PCPs either via emails or in person to participate in the study. The aims of the study were described in the participant information document which accompanied the invitations. Purposive sampling is a sampling method where participants are recruited based on the expectation that each participant will provide unique and rich information of value to the study [24]. Opinions towards acceptance of usage of technology in care of patients may vary among PCPs due to differences in their demographic profiles, training background and type of practices. Primary care practices are variable in scope in Singapore. PCPs can practise in public primary care clinics (polyclinics) or private General Practitioner (GP) Clinics. Selected GP clinics focus solely on aesthetic practices or health screening. Hence, purposive sampling was employed to ensure inclusion of PCPs from different age groups, gender, ethnicity, type of practice and medical training.

### Interviews and transcribing

Demographic data was collected from the participating PCPs via a questionnaire before the interviews, which were conducted either in person or via the teleconferencing platform. The semi-structured topic guide (Supplementary File 1) was sent to the PCPs prior to the interviews to ensure adequate time for them to read and reflect on the stipulated questions. The interviews were carried out by PZM, the principal investigator with approximately 10 years of clinical experience in family medicine and a fellow of the College of Family Physicians, Singapore. Other co-investigators, TNC and ELCW assisted PZM in some of the interviews and took field notes. The IDIs and FGDs were conducted in a private quiet room at the study site. All participants were compensated with SGD20 (approximately USD15) grocery store vouchers as tokens of appreciation for the time devoted to the interviews. The interviews were audio-recorded and professionally transcribed ad-verbatim. The study team then audited and rectified the transcripts to ensure accuracy.

### Coding and analysis

The investigators read the transcripts in full, reflected and coded the qualitative data independently for the first six interviews. Disagreements or differences in the coding were resolved via discussion between the investigators. The codes from these six transcripts were used to finalise the coding frame, which was used to code the remaining nine interviews. Data saturation was defined as the point when no new code emerged [25]. The codes were subsequently grouped into themes and categorised according to the TAM. NVivo software version 12 was used to facilitate the data coding.

## Results

**Fig. 1:**
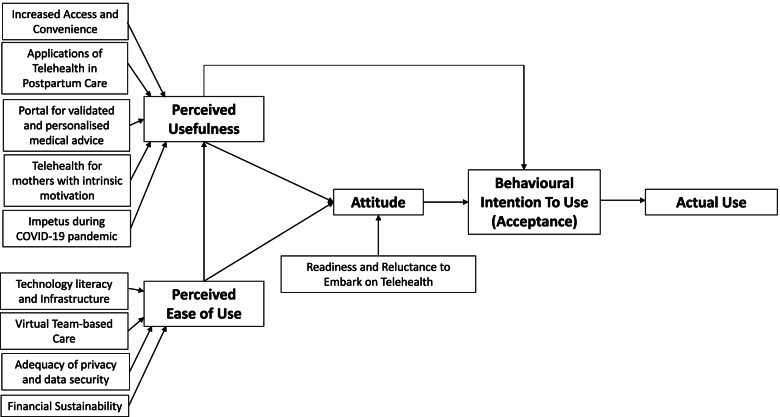
Factors Affecting PCPs’ Acceptance and Use of Telehealth in Postpartum Care using the Technology Acceptance Model (TAM)

29 out of 31 (93.5%) PCPs approached agreed to participate in the study. A total of 4 FGDs and 11 IDIs were conducted. The interviews ranged from 53 to 87 min with an average duration of 69 min. Their demographic characteristics, qualifications and practice profiles are illustrated in Table 1. The themes and subthemes categorised by the TAM are summarized and presented in Fig. 1.

**Table 1:** Characteristics of PCPs interviewed

Characteristics	N (%)
**Age**	
25–34	16 (55.2)
35–44	7 (24.1)
> 45	6 (20.7)
**Gender**	
Male	12 (41.3)
Female	17 (58.6)
**Postgraduate training**	
Bachelor of Medicine and Bachelor of Surgery (MBBS)	9 (31.0)
Graduate Diploma in Family Medicine	4 (13.8)
Master of Medicine (Family Medicine)	11 (37.9)
Fellow of the College of Family Physicians, Singapore	5 (17.3)
**Ethnicity**	
Chinese	24 (82.8)
Malay	1 (3.4)
Indian	1 (3.4)
Others	3 (10.4)
**Type of Practice**	
Private GP practices	5 (17.2)
Public healthcare institutions	24 (82.8)
**Position**	
Medical Officer^a^	4 (13.8)
Resident Physician^b^	4 (13.8)
Family Physician^c^	16 (55.2)
General Practitioner^d^	5 (17.2)

^a^ Qualified physicians granted conditional or full medical registration by the Singapore Medical Council (SMC) without postgraduate qualification

^b^ Qualified physicians with at least an undergraduate medical degree, granted full medical registration and with at least 3 years of clinical experience

^c^ Registered medical practitioners with relevant and recognized postgraduate qualifications (Graduate Diploma in Family Medicine, or a Masters in Family Medicine) and with at least 3 years clinical experience

^d^ Registered medical practitioners with relevant and recognized postgraduate qualifications (Graduate Diploma in Family Medicine or a Masters in Family Medicine) working in the private setting

### Perceived Usefulness

#### Increased access and convenience

Most PCPs agreed that telehealth could play a complementary role to the existing in-person model of postnatal care. This is largely attributed to the convenience it provides to the postpartum mother where medical advice is easily available from the comfort of their home. This virtual consultation could serve the needs of these busy postpartum mothers, who often have multiple domestic chores to commit to, on top of their medical appointments.

*“I guess it is easier for patients. Because they don’t actually have to come and see you with the baby. They may be alone at home. There’s no one to take care of baby. They will be more able to do a tele-consult. Rather than come and see a doctor. Especially if they have many, many children.” (PC24, Female Family Physician)*

#### Complementary to in-person consultation

While telehealth can overcome distance and access, PCPs concurred that it could not replace the current care model. They felt that building relationship and effective communication would be best achieved in person. A postpartum consultation may require the demonstration of hands-on technique, which will not be feasible in telehealth.

*“Yes. If the *keyword* you use is complementary, I agree. Replacement? I don’t think so.” (PC25, Male GP)*

*“It requires a lot of interpersonal relations, especially in the realm of postpartum women whereby there needs to be a form of relationship with the person that you are speaking to, in order for the mother to feel comfortable enough to open up about such issues…it’s still better for face-to-face and some confidentiality.” (PC1, Male Family Physician)*

*“A large group of such patients who actually need help…it’s actually physical help. For example, *breastfeeding* difficulty. A lot of these techniques may need to be demonstrated in person. I don’t know your video can do it? I don’t know. You want to flash on the video? [laughs]” (PC4, Female Family Physician)*

#### Specific advantages of telehealth

PCPs specified the applications of telehealth in postnatal care, including mental health screening of postpartum women, who may require closer follow-up or referral to early interventional therapy. Additionally, telehealth provides women with early access to scientific information on normal baby behaviour and the skills and knowledge in the care of the newborn. This information can be delivered either in the form of oral advice or referral to digital or written resources. The telehealth provider can be trained paramedical staff, such as nurses or midwives.

*“Telehealth can be performed by the nurse to check on maternal well-being. With the telehealth service that they pick up postnatal blues or postnatal depression, they can refer the mummy to other services or ask the mummy to come back to the polyclinic for us to do the assessment… Where we can do screening, we can reach out to a lot of patients” (PC2, Female Family Physician)*

*“if you’re talking about coaching, in terms of how to wean your child, how to sleep-train your child, how to breastfeed, how to relieve a latching problem, I think, it can be very helpful.” (PC22, Male Medical Officer)*

#### Portal to provide validated and personalised medical advice

Some PCPs cited the availability of advice on care of the newborn in the community, such as those from the women’s family and friends, online forums, religious and other support groups. However, the postpartum mother often has to plough through immense volumes of information from these sources, which occasionally may not be correct, or even be conflicting, resulting in confusion instead. A telehealth service manned by well-trained medical personals would enable mothers to clarify their individual doubts and receive information that is validated, evidence-based and trustable.

*“These resources (from telehealth provider) are sort of more certified, because it comes from a *healthcare* personnel.” (PC6, Male Resident Physician)*

*“..if they (mothers) have any concern, they can just text it and ask and get a professional kind of advice, instead of some advice by other mummies.” (PC10, Female Resident Physician)*

*“It will be *good* for a professional to give accurate advice.” (PC20, Female Family Physician)*

#### Telehealth for selected group of mothers with intrinsic motivation

The PCPs differed on the profile of postpartum mother whom they perceived to benefit from the telehealth service. While most of them agreed in principle that telehealth should be offered to all postpartum mothers, some of them pointed out it suited mothers who were motivated and keen to seek medical advice from medical professionals.

*“…there will be a group of patients who are more resistant to telehealth…they may be the ones that actually need more help, but not SEEKING (emphasised) the help. So, if they are not SEEKING *the* help, then you offer them a telecare or telehealth system, (it is) unlikely that they will want to engage this system.” (PC3, Female Family Physician)*

*“…probably more acceptable to those who are very motivated, educated and they actually want to know more…for the other half of the women who will just follow whatever their mother says, *whatever* their mother-in-law says, this support will be a bit redundant because there might be a conflict in the knowledge between these two groups, she’ll be very confused.” (PC21, Female Family Physician)*

#### Impetus during the Covid-19 pandemic

PCPs believed the current COVID-19 pandemic would accelerate the adoption of this care model among postpartum women. The latter might worry about the well-being of their vulnerable newborn in a healthcare facility due to potential cross-infection with the coronavirus.

*“…time pressure on women, and also the COVID period. The fear of having to lug their baby out to the clinic just to see the doctor. So yes, I think it’s especially applicable to mothers.” (PC23, Female GP)*

### Perceived Ease of Use

#### Technology literacy and infrastructure

Most PCPs shared their observation that telehealth adoption by mothers would be gradual, even in a highly digitalized country with prevalent use of mobile technology. This is due to the perceived preference for physical consults for more personal issues during the postpartum period. However, PCPs believed that the high information technology (IT) and digital health literacy of the population, especially amongst the younger generation, would facilitate their acceptance and utilisation of the service.

*“The more tech-savvy sort of generation, people are going online all the time, it should be a growing acceptance (of telehealth)…but initially I think particularly when it comes to this period of their life, I think there’s a lot of sensitivity and there may be still a preference to actually SEE the person, to be able to connect, you know, to receive the support and things like that… the reliance on some of the telehealth support, right, I suppose, will grow over the years lah maybe, you know, but as a start, we have still 60% or 70% of the postpartum care delivered onsite, and then you have, you know, maybe 20% that can be delivered by this, and then slowly you grow.” (PC14, Male Family Physician)*

#### Virtual team-based care for post-natal mothers

The PCPs identified trained human resource availability as an enabler to enhance the utilisation and successful implementation of the telehealth-based model of postpartum care. The local public primary care system is adopting team-based care as key to managing an aging population with complex healthcare needs. PCPs suggested setting up similar team-based postpartum care involving other paramedical and allied health professionals such as lactation consultants in providing the telehealth service. The different domain experts could value-add to provide comprehensive and personalised care for the mother and complement the care delivery by the PCPs.

*“You probably need a lactation consultant on board to help with the breastfeeding issues…we can have all the other nurses also involved and see (manage the mothers).” (PC22, Male GP)*

#### Balance workload and access to specialists for support

Several PCPs pointed out to the adequacy of manpower to operationalize the telehealth-based care model. They were concerned about additional workload and extended working hours to support the proposed telehealth-based care model.

“The question is how do we actually provide that service while the doctor tries to stick to his hours; whether or not it’s going to off-hours kind of arrangement.” (PC22, Male GP, employee)

*“…some doctors, especially people practising in primary care… we have… office hours and *all* that, right, so people must be available beyond office hours, so this is … barrier for some of the doctors practising in primary care.” (PC14, Male Family Physician)*

PCPs perceived the need to access specialists in tertiary institution to manage cases beyond their expertise. Such additional support would lower their perceived medicolegal risk in providing care for mothers over telehealth.

*“…am I sufficiently *supported*? Because for postpartum care, a well-trained family physician can handle 70–80% of the issues. Now, for the 10–20%, if there is a need for referral…Am I supported in giving the parents, the new parents the channel? The medical legal risk for telemedicine is… tele-advice, tele-support, tele-consult, it’s not that clear cut.” (PC25, Male GP)*

#### Adequacy of Privacy and data security

Other serious concern highlighted by PCPs were related to privacy and data security due to potentially sensitive images and video recorded over the telehealth platform. They were concerned about protecting such data from cyberattacks and unauthorised access and receptiveness of mothers towards such a functionality over the telehealth platform.

*“People may not be comfortable, because of concerns about hacking or photos of their breast being seen by others.” (PC23, Female GP)*

*“I mean if you are actually showing a real breast over video-consult… is it being recorded? Is this *safe*? So there’s no element of safety, I’m not sure how the patients would be receptive to it.” (PC24, Female Family Physician)*

#### Financial sustainability in a pay-for-service primary healthcare system

PCPs viewed financial sustainability as key to successful implementation of the new care model. They related it to adequate subvention from the government in the public polyclinics and acceptable remuneration for the private GPs.

*“If you use the current telemedicine vendors…Their charge is only about $10-$20 per consult. I don’t think *that* is sufficient for a senior family physician that is well trained in postpartum care.” (PC25, Male GP)*

*“One important discussion would be with of course the ministry, to see how much they can support by subvention, to make sure that we at least don’t run a loss running the service, so go by that fee.” (PC14, Male Family Physician)*

*“First of all, like at the end of the day, we are … a business…we want to make sure whatever we’re doing, we’re generating revenue…we want to make sure that we’re remunerated fairly *for* the amount of work that we put in.” (PC22, Male GP)*

PCPs highlighted that the cost of the telehealth postnatal service would influence the users’ (mothers’) acceptance and its utilisation. Most PCPs perceived benefits for the postpartum women, but anticipated their unwillingness to pay for such a service.

*“We like “cheap, fast, good”. So, we want cheap. This one (telehealth) is fast and good, but is *not* cheap, I don’t think Singaporeans will want.” (PC6, Male Resident Physician)*

*“I mean, it’s really good, but if you are asking patients to pay for this, I think your response rate (*acceptance* of telehealth service) might be dismal.” (PC16, Male Family Physician)*

### Attitude

#### Readiness and reluctance to embark on telehealth

The PCPs revealed variable levels of enthusiasm and willingness in telehealth-based postpartum care. They felt that those with special interest in maternal and child health and early adopters of telehealth could try out the new care model.

*“I think doctors who are interested in postpartum care, maternal care would be happy to helm this because it is beneficial to women. But for those who are not comfortable seeing postpartum women or being involved in postpartum care, they may not be comfortable, especially since this is a teleconsult model.” (PC23, Female GP)*

Older PCPs appeared reluctant to accept this novel model of care due to concerns about competition from younger telehealth providers. They were worried that mothers enrolled into telehealth would stop seeking in-person consultation with them.

*“Okay, 20 years down the road, maybe everybody will be on the platform. All PCPs will be on the platform. But if you ask me today, that 70% very unwilling people will not be on that platform. And that very 70% oldies, I told you the bracket of people who are not keen on that, will *not* be in. They are the people who are wondering where their patients went to. And then it’s suddenly, going to this er…postnatal…some fancy name that you give to this platform, specifically for postpartum. And you find it being helmed by Dr…, you know all the young doctors, then who are these people taking away my patients!” (PC26, Female GP)*

## Discussion

This study explored acceptance of telehealth among PCPs when delivering postpartum care. The use of the TAM allows us to understand users’ acceptance and highlight potential challenges before they interact with the system [26]. The PCPs’ acceptance depended on their perceived usefulness and ease of use of telehealth in addressing the care needs of the postpartum mother, and their proposed solutions to overcome the multiple challenges.

### Perceived usefulness

Telehealth has been increasingly used by PCPs to provide medical care to patients before and especially during the pandemic. The usage of telehealth to complement in-person primary care services has been largely met with positive responses [18, 27]. A systematic review has reported the effectiveness of telehealth interventions in improving maternal health outcomes related to breastfeeding and continuation of their contraception after their delivery [17]. In this study, the PCPs have also recognised the merits and usefulness of the proposed telehealth-based postpartum care model. They have acknowledged the main draw of telehealth services in primary care, which include convenience, reduction of in-person visits to mitigate cross-infection amidst the pandemic [28].

The extension of telehealth to postpartum care can potentially expand the capacity of the PCP in meeting the healthcare needs of the mothers at points of time when support is most required. The multitude of health issues and concerns in the postpartum period can be overwhelming to address in a single postpartum visit to a PCP [29]. Incorporating telehealth-based consultation soon after the birth of the child allows earlier maternal assessment. Additional in-person consultation can be arranged, pending on specific needs of the mother. Such a hybrid care model will align to the WHO guidelines which recommend at least three postnatal contacts for mothers [2].

The engagement of mothers to this hybrid postpartum care model should begin early, even during the antenatal period. Sunny S et al. had shown inconsistent hand-over of postpartum care of mothers from the obstetricians to PCPs. The study further suggests that mothers could be better educated on postnatal care issues during their pregnancy via antenatal programmes [30]. PCPs can promote the hybrid postpartum care model to mothers attending these antenatal programmes. During these interactions, feedback regarding the acceptability of telehealth can be gathered simultaneously. This approach allows PCPs to establish a relationship prior to delivery and provide an avenue for mothers to seek professional help for their needs in the postpartum period. Payment to utilise this enhanced service may become less of a barrier if mothers are aware of the purpose and value and reduce the financial concerns alluded by the PCPs in this study.

### Perceived ease of use

Up to 88% of the population in Singapore own and use a mobile phone [31]. A study in Singapore showed that majority of the population, owning a mobile phone, were interested in mobile health services [32]. This is in line with the PCPs’ perceived high acceptance rate of telehealth among postpartum mothers. The digitalisation of medical records implemented in all polyclinics and the majority of private PCPs [33] can further facilitate continuity of care as it allows ready access by PCPs during video consultations with postpartum mothers.

Optimization of this proposed telehealth care model hinges on the support from institutions and specialist partners, which the PCPs highlighted as important determinants for its success. A systematic review has showed that collaboration between specialists and PCPs may confer modest long-term health benefits through improvements in patient concordance with treatment programmes and more effective clinical practice [34]. Locally, a variant collaborative care model between PCPs, multidisciplinary primary care teams and specialists from tertiary hospitals has succeeded in providing cognitive assessment service to patients in the polyclinics [35]. Similarly, obstetricians can work together with PCPs to develop an integrated maternal and child health service where postpartum care training and specialist support for video consultation are provided. This will empower the PCPs in their clinical and technical competencies regarding postpartum care management.

Lack of time for primary care consultations is common, even more so for a postpartum consultation which can easily take up to 25 min as reported in an Australian study on local GPs [36]. In contrast, the average duration of a GP consultation was 9.3 min in a Singapore study [37]. The limited consultation time is likely to adversely affect care delivery and physician workload, resulting in stress and burnout [38]. Utilisation of validated electronic questionnaire and other tools to triage and identify key agenda prior to the video-consultation and involvement of a multidisciplinary team are ways to streamline the service, so that the main issues are adequately addressed within a single telehealth consult despite the limited time. As mentioned by the PCPs, support from the institution leadership in allocating sufficient resources to support the hybrid care model and train the PCPs is pivotal to ease its implementation and enhance the well-being of the primary care providers.

The Covid-19 pandemic has greatly accelerated the progression of development of telehealth platforms in both the private and public sector. However, an important consideration is the security of the data transmitted digitally. There have been incidences of major breach of personal data in Singapore’s healthcare system in recent years [39, 40]. A systematic review showed that the healthcare industry lags behind in security which makes it a prime target for medical information theft [41]. Policy makers and healthcare institutions will need to institute proper measures for cybersecurity. These include investing in risk management systems and training employees to recognise and be vigilant against cyberattacks [42]. Nonetheless, implementation of risk management systems may impact on care of patients as access to patient information by care providers across healthcare institutions may be slowed down or denied due to safety measures in place. These factors will need to be deliberated by our policy makers.

### Attitude

Adoption of novel healthcare technology and use of new care model varies between healthcare providers [43]. Change management for telehealth service implementation takes time, and the attitude of stakeholders matters as it determines their readiness to adopt and adapt to changes [44]. Their attitude in turn can be influenced by multiple personal factors such as age, background education or subspecialty [45]. In this study, a senior PCP appeared less ready for telehealth services, perhaps at a stage where retirement takes priority over investment of time and resources to set up new care model.

In addition, the PCPs’ attitude and interest in telehealth appears to be related to the estimated remuneration from operating such a service [46]. Telehealth implementation can improve accessibility to on-demand virtual medical consultations via electronic devices and increase convenience of access for the mothers from the comfort of their homes. These factors can potentially lead to excess use and drive up healthcare costs [47]. Significant changes to the local healthcare financial system seem inevitable to sustain the newer telehealth-based services and reimburse the providers. The current fee-for-service healthcare system [8] has invariably impacted on the adoption and scaling of telehealth services, as it relies on the willingness of the end-users to pay for such services. Feasibility study is thus essential for a comprehensive evaluation on the viability of setting up a hybrid care model for postpartum care. A successful used case amidst a threatening pandemic-stricken healthscape may modify the attitude of the PCPs and increase its adoption.

### Strengths and limitations

The TAM has been widely used to evaluate user acceptance, highlight potential design gaps and predict adoption of novel healthcare technology. This theoretical framework embedded in the qualitative research methodology of this study enables the exploration of inter-related personal, patient and healthcare system factors, which are postulated to influence the PCPs’ acceptance of telehealth for postpartum care. Under the ‘perceived ease of use’ domain in TAM, the barriers to PCPs adopting telehealth are presented as the difficulties to use telehealth (e.g. lack of development of team based care, potential for data hacking and doubt regarding financial sustainability).

Our study has its limitations. The data collected from selected participants in this study are not generalizable to the local PCP population. However, purposive sampling was carried out in order to identify a wide range of factors influencing PCPs’ views on telehealth in postpartum care.

While the qualitative study allows us to identify the multitude of factors, there is no quantitative measure of the weight of each factor to prioritise interventions. However, addressing most of these hurdles alluded by the PCPs will increase the probability of successful implementation of the hybrid care model and concurrently safeguard the interests of the PCPs.

More public PCPs were recruited in this study than private GPs, which may potentially skew the results. Nonetheless, the public PCPs (and not the obstetricians) are likely the major healthcare provider for postpartum mothers. Aside from obstetricians who deliver their infants, mothers are more likely to consult the public PCPs during the immediate postpartum period (e,g, in the case of neonatal jaundice) due to availability of in-house laboratory services at the polyclinics. There appears to be a relatively large number of PCPs of Chinese ethnicities recruited. However, 74.6% of the population in Singapore are Chinese, which is comparable to the purposively sampled population in our study [48].

Gathering the perspectives of postpartum mothers on their acceptability of the proposed care model is equally important as the views of the PCPs in this study. The investigators have completed a parallel qualitative research study to identify the needs of the postpartum mothers and garner their views on the hybrid care model. The results will be presented separately.

## Conclusion

The study showed that most PCPs perceived usefulness and ease of use of video consultation in delivering postpartum care. They recognised that telehealth service would complement and support the current face-to-face postpartum care for mothers amidst the pandemic. However, training, leadership support, and organizational infrastructure, healthcare financial policy and personal demographic profile influence and shape their attitude. These are important determinants of their acceptance and utilisation of the proposed hybrid (telehealth and in-person) postpartum care model.

## Supplementary Information

**Additional file 1.**

## Data Availability

The data used to support the findings of this study are included within the article. Raw data analysed during the study are not publicly available due to confidentiality agreements but can be made available from the corresponding author on reasonable request.
