# Supplementary material for: A qualitative research study of primary care physicians’ views of telehealth in delivering postnatal care to women
Source: BMC Prim Care. 2022 Aug 13;23:206. doi: 10.1186/s12875-022-01813-9 (PMC9375064; doi:10.1186/s12875-022-01813-9)
Supplement: Supplementary file 1 — Additional file 1. [file 12875_2022_1813_MOESM1_ESM.docx]

**Supplementary File 1**

| **Supplementary File 1. Interview Guide** |
| --- |
| 1. What do you understand of postpartum care and how long it spans? 2. What are your views regarding the role of primary care physicians in providing postpartum care for women? 3. What is your approach to assess postpartum needs of women? 4. What are the resources that are available for you to better manage these women? 5. How do you think postpartum care can be improved in the primary care sector? 6. What are your views towards telehealth (to show participants a picture of what telehealth consists of) as a new model of care to compliment the current delivery of postpartum care in the primary care setting?   Detailed questions as guided by the TAM:  Perceived usefulness   - How do you think this can benefit and apply to your setting of practice? - What are the gaps in postpartum care that telehealth can fill? - What are the limitations of telehealth in postpartum care?   Perceived ease of use   - How feasible do you think telehealth is for our local population? - Would mothers be open to this model of care? - What are the resources needed for the implementation of this care? Is it adequate in your setting?   Attitude   - Would doctors and other medical personnel to open to this model of care? - Which medical personnel may be suitable to provide the care? |
